# Phylogenetic Relationships of *Citrus* and Its Relatives Based on *matK* Gene Sequences

**DOI:** 10.1371/journal.pone.0062574

**Published:** 2013-04-25

**Authors:** Tshering Penjor, Masashi Yamamoto, Miki Uehara, Manami Ide, Natsumi Matsumoto, Ryoji Matsumoto, Yukio Nagano

**Affiliations:** 1 Department of Applied Biological Sciences, Saga University, Honjo, Saga, Japan; 2 Renewable Natural Resources Research Centre Wengkhar, Mongar, Bhutan; 3 Faculty of Agriculture, Kagoshima University, Korimoto, Kagoshima, Japan; 4 Analytical Research Center for Experimental Sciences, Saga University, Honjo, Saga, Japan; United States Department of Agriculture, United States of America

## Abstract

The genus *Citrus* includes mandarin, orange, lemon, grapefruit and lime, which have high economic and nutritional value. The family Rutaceae can be divided into 7 subfamilies, including Aurantioideae. The genus *Citrus* belongs to the subfamily Aurantioideae. In this study, we sequenced the chloroplast *matK* genes of 135 accessions from 22 genera of Aurantioideae and analyzed them phylogenetically. Our study includes many accessions that have not been examined in other studies. The subfamily Aurantioideae has been classified into 2 tribes, Clauseneae and Citreae, and our current molecular analysis clearly discriminate Citreae from Clauseneae by using only 1 chloroplast DNA sequence. Our study confirms previous observations on the molecular phylogeny of Aurantioideae in many aspects. However, we have provided novel information on these genetic relationships. For example, inconsistent with the previous observation, and consistent with our preliminary study using the chloroplast *rbcL* genes, our analysis showed that *Feroniella oblata* is not nested in *Citrus* species and is closely related with *Feronia limonia*. Furthermore, we have shown that *Murraya paniculata* is similar to *Merrillia caloxylon* and is dissimilar to *Murraya koenigii*. We found that “true citrus fruit trees” could be divided into 2 subclusters. One subcluster included *Citrus*, *Fortunella*, and *Poncirus*, while the other cluster included *Microcitrus* and *Eremocitrus*. Compared to previous studies, our current study is the most extensive phylogenetic study of *Citrus* species since it includes 93 accessions. The results indicate that *Citrus* species can be classified into 3 clusters: a citron cluster, a pummelo cluster, and a mandarin cluster. Although most mandarin accessions belonged to the mandarin cluster, we found some exceptions. We also obtained the information on the genetic background of various species of acid citrus grown in Japan. Because the genus *Citrus* contains many important accessions, we have comprehensively discussed the classification of this genus.

## Introduction

The genus *Citrus*, which includes mandarin, orange, lemon, grapefruit and lime, has high economic and nutritional value. This genus belongs to the subfamily Aurantioideae, which is one of the 7 subfamilies of the family Rutaceae. Therefore, phylogenetic study of both the genus *Citrus* and of the subfamily Aurantioideae is important.

The Aurantioideae consists of 2 tribes with 33 genera [1]. These 2 tribes are each composed of 3 subtribes: the tribe Clauseneae, which includes Micromelinae, Clauseninae, and Merrillinae; and the tribe Citreae, which includes Triphasiinae, Citrinae, and Balsamocitrinae. None of the Clauseneae species develop axillary spines, and the odd-pinnate leaves have alternately attached leaflets. The fruits are usually small and carry semi-dry or juicy berries, except in *Merrillia*. In contrast, nearly all the species develop axillary spines in the Citreae. The simple leaves are easily distinguished from those of the tribe Clauseneae. The subtribe Citrinae, in the tribe Citreae, is distinct from all the other subtribes in the Aurantioideae because of the presence of pulp vesicles in the fruit. In this subtribe, “true citrus fruit trees” are considered the most advanced genera based on morphological traits [1]. The genus *Citrus* belongs to the “true citrus fruit trees.” The characteristics of *Citrus* species include asexual reproduction, high mutation frequency, and cross compatibility between species. Because of these characteristics, there is great morphological and ecological diversity among *Citrus* species.

Since the 1970s, morphological [2]–[4] and biochemical studies [5]–[9] have been conducted to elucidate the phylogeny of Aurantioideae, especially of *Citrus* and its close relatives. Because of improved DNA analysis, these relationships have been studied extensively. Several techniques, such as restriction fragment length polymorphism (RFLP), random amplified polymorphic DNA (RAPD), simple sequence repeat (SSR), and sequence-related amplified polymorphism (SRAP) have been commonly used in taxonomic studies [10]–[14].

Recent progress in DNA sequencing techniques has allowed the extensive use of short DNA fragments, especially those of the chloroplast genome, in the study of phylogenetic relationships. Phylogenetic analyses based on the various regions of the chloroplast genome have been conducted in the family Rutaceae and the subfamily Aurantioideae [15]–[24].

We have also previously reported the phylogenetic relationships among the Aurantioideae, including *Citrus* and its relatives, based on *rbcL* gene sequences [25]. The *rbcL* gene, located on the chloroplast DNA (cpDNA), encodes the large subunit of ribulose 1, 5-bisphosphate carboxylase/oxygenase, an enzyme that catalyzes carbon fixation in photosynthesis. Compared to most genes encoded in the cpDNA, the *rbcL* gene has a relatively slow nucleotide substitution rate [26]–[28]. A characteristic feature of our previous study [25] is that it included several accessions, which had not been examined in other studies [15]–[24]. However, the power of discrimination in our previous study was not high.

The *matK* gene is also located on the cpDNA and encodes a maturase involved in splicing type II introns from RNA transcripts. The *matK* gene is encoded by the chloroplast *trnK* intron. Since *matK* has a relatively fast mutation rate, it evolves faster than the *rbcL* gene [26]–[28]. Therefore, *matK* analysis should be useful for studying the phylogeny of the genera included in Aurantioideae.

To comprehensively analyze the phylogenetic relationships of the superfamily Aurantioideae, we determined the *matK* sequences of 135 accessions from 22 genera of the Aurantioideae. In this study, we used *matK* sequences derived from basic and major species of the Aurantioideae. Similar to our previous study [25], our current study included several accessions that had not been examined in other studies [15]–[24]. Furthermore, we increased the number of accessions that were analyzed, in order to focus on interspecific relationships among the mandarin varieties of the *Citrus* genus because several studies have suggested the existence of great genetic variation among mandarin varieties [5], [6], [29], [30]. Our study included many kinds of mandarin varieties grown in Japan, many of which have not been studied previously at the DNA level. The genetic background of various species of acid citrus (considered to be of hybrid origin) grown in Japan has also been investigated.

## Materials and Methods

### Plant Materials

The 135 accessions from 22 genera of the Rutaceae subfamily Aurantioideae that were used in this study as well as the sources of the materials are shown in Tables 1 and 2. The materials have been preserved at the Faculty of Agriculture, Saga University, the Saga Prefectural Fruit Tree Experimental Research Station, the Faculty of Agriculture, Kagoshima University, and the National Institute of Fruit Tree Science.

**Table 1:** Species belonging to Aurantioideae (excluding *Citrus)* used in this study.

Tribe	Subtribe	Group	Latin name (Common name)	No.^z^
Clauseneae	Micromelinae		*Micromelum minutum* (Forst.) Wt. & Arn.	8650
	Clauseninae		*Clausena anisata* (Willd.) Hook. f.	8612
			*C. harmandiana* (Pierre) Guill.	8613
			*C. lansium* (Lour.) Skeels	8611
			*Glycosmis citrifolia* (Willd.) Lindl.	8601
			*G. pentaphylla* (Retz.) Correa	8600
			*Murraya koenigii* (L.). Spreng.	8622
			*M. paniculata* (L.) Jack.	8621
			*Merrillia caloxylon* (Ridl.) Swing.	8640
Citreae	Triphasinae	Triphasia group	*Paramignya lobata* Burkill	8350
			*Triphasia trifolia*(Burm. f.) P. Wils. f.	8500
	Citrinae	Primitive citrus fruit trees	*Hesperethusa crenulata* (Roxb.) Roem.	8320
			*Severinia buxifolia* (Poir.) Tenore	8340
		Near citrus fruit trees	*Atalantia bilocularis* (Roxb.) Wall. ex Skeels	8312
			*A. ceylanica* (Arn.) Oliv.	8310
			*A. monophylla* DC.	8314
			*A. roxburghiana* Hook. f.	8316
			*A. spinosa* (Willd.) Tanaka	8315
			*Citropsis gabunensis* (Engl.) Swing. & M. Kell.	8300
			*C. gilletiana* Swing. & M. Kell.	8302
			*C. schweinfurthii* (Engl.) Swing. & M. Kell.	8301
			*Clymenia polyandra* (Tan.) Swing.	8280
			*Eremocitrus glauca* (Lindl.) Swing.	8251
			*Fortunella crassifolia* Swing. (meiwa kumquat)	8000
			*F. hindsii* (Champ.) Swing. ‘Diploid (2x)’ (Hongkong wild kumquat)	8004
			*F. hindsii* (Champ.) Swing. ‘Tetraploid (4x)’	8005
			*F. japonica* (Thunb.) Swing.	8002
			*F. margarita* (Lour.) Swing. (oval kumquat)	8001
			*F. obovata* hort. ex Tanaka (changshou kumquat)	8003
			*F. polyandra* (Rindl.) Tanaka (Malayan kumquat)	8006
			*Microcitrus australasica* (F. Muell.) Swing.	8203
			*M. australis* (Planch.) Swing.	8201
			*M. inodora* (F. M Bail.) Swing.	8209
			*M. papuana* H. F. Winters	9210
			*M. warburgiana* (F. M. Bail.) Tanaka	8206
			*Poncirus trifoliata* (L.) Raf. ‘Flying Dragon’ (trifoliate orange)	8103
			*P. trifoliata* (L.) Raf. ‘Standard’ (trifoliate orange)	8100
	Balsamocitrinae	Tabog group	*Swinglea glutinosa* (Blanco) Merr	8420
		Bael fruit group	*Aegle marmelos* (L.) Corr.	8400
			*Afraegle paniculata* (Schum.) Engl.	8411
		Wood apple group	*Feronia limonia* (L.) Swing.	8411
			*Feroniella oblata* Swing.	8460
Outgroup			*Zanthoxylum* sp. Clayton 15	–

z Accession number at the Saga University.

**Table 2:** *Citrus* species and accessions used in this study.

	No.^z^	Latin name		Common name	Accession	Source^x^	Accession
		Tanaka^z^	Swingle and Reece^y^				No.
ArchicitrusPapeda							
	1	*Citrus macroptera* Montr.	*Citrus macroptera* Montr.	Melanesian papeda		A	7006
	4	*C. micrantha* Wester.	*C. micrantha* Wester.	Biasong		A	7004
	7	*C. hystrix* DC.	*C. hystrix* DC.	Mauritius papeda		A	7003
	10	*C. latipes* (Swing.) Tanaka	*C. latipes* (Swingle) Tanaka	Khasi papeda		A	7000
Citron and its relatives							
	13	*C. aurantifolia* (Cristm.) Swing.	*C. aurantifolia* (Cristm.) Swing.	Lime	Mexican	A	5115
	15	*C. latifolia* Tanaka	*C. aurantifolia* (Cristm.) Swing.	Bearss lime		B	9432
	16	*C. limettioides* Tanaka	*C. aurantifolia* (Cristm.) Swing.	Sweet lime		B	5638
	31	*C. medica* L.	*C. medica* L.	Citron	Maru Busshukan	A	5001
	31	*C. medica* var. sarcodactylis Swing.	*C. medica* var. sarcodactylis Swing.	Citron	Fingered Citron	A	5008
	36	*C. limon* (L.) Burm. f.	*C. limon* (L.) Burm. f.	Lemon	Eureka	C	–
	36	*C. limon* (L.) Burm. f.	*C. limon* (L.) Burm. f.	Lemon	Villafranca	A	5207
	39	*C. jambhiri* Lush	*C. limon* relative	Rough lemon		B	–
Pummelo and its relatives							
	56	*C. maxima* (Burm.) Merr.	*C. maxima* (Burm.) Merr.	Pummelo	Mato Buntan	A	3202
	56	*C. maxima* (Burm.) Merr.	*C. maxima* (Burm.) Merr.	Pummelo	Suisho Buntan	A	3301
	56	*C. maxima* (Burm.) Merr.	*C. maxima* (Burm.) Merr.	Pummelo	Banpeiyu	A	3206
	78	*C. natsudaidai* Hayata	*C. maxima* hybrid	Natsuidaidai	Kawano Natsudaidai	B	–
	79	*C. obovoidea*hort. ex Takahashi	*C. maxima* hybrid	Kinkoji			
Sour and sweet oranges and their relatives							
	93	*C. aurantium* L.	*C. aurantium* L.	Sour orange	Kabusu	D	JP117365
	93	*C. aurantium* L.	*C. aurantium* L.	Sour orange	Zadaidai	C	–
	94	*C. myrtifolia* Rafin	*C. aurantium* L.	Myrtle-leaf orange		C	–
	99	*C. canaliculata* hort. ex Y. Tanaka	*C. aurantium* relative	Kikudaidai		B	–
	100	*C. sinensis* (L.) Osbeck	*C. sinensis* (L.) Osbeck	Sweet orange	Fukuhara	A	2100
	100	*C. sinensis* (L.) Osbeck	*C. sinensis* (L.) Osbeck	Sweet orange	Valencia	A	2200
	103	*C. tankan* Hayata	*C. sinensis* hybrid	Tankan	Tarumizu 1-Gou	C	-
	-	*C*. hybrid cultivar	*C. aurantium* relative	Fuiri daidai		B	–
MetacitrusYuzu and its relatives							
	112	*C. ichangensis* Swing.	*C. ichangensis* Swing.	Ichang papeda		A	7009
	113	*C. junos* Siebold ex Tanaka	*C. ichangensis* relative	Yuzu	Yamane	A	5403
	113	*C. junos* Siebold ex Tanaka	*C. ichangensis* relative	Yuzu	Touchikei	A	5404
	113	*C. junos* Siebold ex Tanaka	*C. ichangensis* relative	Yuzu	Tetraploid Yuzu	A	5402
	113	*C. junos* Siebold ex Tanaka	*C. ichangensis* relative	Yuzu	Tadanishiki	B	–
	114	*C. hanaju* hort. ex Shirai	*C. ichangensis* relative	Hanaju		A	5500
	115	*C. sudachi* hort. ex Shirai	*C. ichangensis* relative	Sudachi	Mushi Yukaku	A	5511
	115	*C. sudachi* hort. ex Shirai	*C. ichangensis* relative	Sudachi	Sudachi	A	5501
	115	*C. sudachi* hort. ex Shirai	*C. ichangensis* relative	Sudachi	Yushi Mukaku	A	5512
	116	*C. inflata* hort. ex Tanaka	*C. ichangensis* relative	Mochiyu		A	5521
	117	*C. yuko* hort. ex Tanaka	*C. ichangensis* relative	Yuko	Yuko	A	5525
	117	*C. yuko* hort. ex Tanaka	*C. ichangensis* relative	Yuko	Mukaku Yuko	A	5526
	120	*C. wilsonii* Tanaka	*C. ichangensis* relative	Ichang lemon		B	0611
	121	*C. sphaerocarpa* hort. ex Tanaka	*C. ichangensis* relative	Kabosu		A	5503
Mandarin							
	123	*C. nobilis* Lour.	*C. reticulata* Blanco	Kunenbo		C	–
	123	*C. nobilis* Lour.	*C. reticulata* Blanco	King		A	1522
	124	*C. unshiu* Marcow.	*C. reticulata* Blanco	Satsuma mandarin	Original strain	A	1300
	124	*C. unshiu* Marcow.	*C. reticulata* Blanco	Satsuma mandarin	Aoshima Unshiu	A	1401
	124	*C. unshiu* Marcow.	*C. reticulata* Blanco	Satsuma mandarin	Imamura Unshiu	A	1403
	124	*C. unshiu* Marcow.	*C. reticulata* Blanco	Satsuma mandarin	Jutaro Unshiu	A	1415
	124	*C. unshiu* Marcow.	*C. reticulata* Blanco	Satsuma mandarin	Sasebo Unshiu	A	1322
	125	*C. yatsushiro* hort. ex Tanaka	*C. reticulata* Blanco	Yatsushiro		D	JP117388
	126	*C. keraji* hort. ex Tanaka	*C. reticulata* Blanco	Keraji		A	1542
	126	*C. keraji* hort. ex Tanaka	*C. reticulata* Blanco	Kabuchii		A	1541
	127	*C. oto* hort. ex Y. Tanaka	*C. reticulata* Blanco	Oto		A	1540
	128	*C. tarogayo* hort. ex Y. Tanaka	*C. reticulata* Blanco	Tarogayo		A	6000
	128	*C. tarogayo* hort. ex Y. Tanaka	*C. reticulata* Blanco	Unju		C	–
	130	*C. reticulata* Blanco	*C. reticulata* Blanco	Ponkan	Yoshida Ponkan	C	–
	131	*C. deliciosa* Ten.	*C. reticulata* Blanco	Mediterranean mandarin		D	JP117393
	132	*C. genshokan* hort. ex Tanaka	*C. reticulata* Blanco	Genshokan		C	–
	133	*C. tangerina* hort. ex Tanaka	*C. reticulata* Blanco	Dancy		D	JP117396
	134	*C. clementina* hort. ex Tanaka	*C. reticulata* Blanco	Clementine		C	–
	134	*C. clementina* hort. ex Tanaka	*C. reticulata* Blanco	Clementine	Clementine de Nules	B	5653
	137	*C. platymamma* hort.ex Tanaka	*C. reticulata* Blanco	Binkitsu		B	–
	140	*C. suhuiensis* hort. ex Tanaka	*C. reticulata* Blanco	Shikaikan		A	1584
	143	*C. tachibana* (Makino) Tanaka	*C. tachibana* (Makino) Tanaka	Tachibana		C	–
	145	*C. kinokuni* hort. ex Tanaka	*C. reticulata* Blanco	Kinokuni	Sakurajima Komikan	C	–
	145	*C. kinokuni* hort. ex Tanaka	*C. reticulata* Blanco	Kinokuni	Hirakishu	A	1518
	145	*C. kinokuni* hort. ex Tanaka	*C. reticulata* Blanco	Kinokuni	Mukaku Kishu	A	1519
	145	*C. kinokuni* hort. ex Tanaka	*C. reticulata* Blanco	Soukitsu		D	JP117400
	148	*C. sunki* (Hayata) hort. ex Tanaka	*C. reticulata* Blanco	Sunki		C	–
	149	*C. reshni* hort. ex Tanaka	*C. reticulata* Blanco	Cleopatra		C	–
	153	*C. depressa* Hayata	*C. tachibana* relative	Shiikuwasha	Okitsu strain	C	–
	153	*C. depressa* Hayata	*C. tachibana* relative	Shiikuwasha	Kabishi	A	1908
	153	*C. depressa* Hayata	*C. tachibana* relative	Shiikuwasha	Mikanguwa	A	1916
	153	*C. depressa* Hayata	*C. tachibana* relative	Shiikuwasha	Fusubuta	A	1911
	153	*C. depressa* Hayata	*C. tachibana* relative	Shiikuwasha	Kaachi	A	1909
	153	*C. depressa* Hayata	*C. tachibana* relative	Shiikuwasha	Ishikunibu	A	1910
	153	*C. depressa* Hayata	*C. tachibana* relative	Shiikuwasha	Shiikunin	C	–
	153	*C. depressa* Hayata	*C. tachibana* relative	Shiikuwasha	Shiikuribu	C	–
	154	*C. leiocarpa* hort. ex Tanaka	*C. reticulata* Blanco	Koji		C	–
	–	*C*. hybrid cultivar	*C. reticulata* Blanco	Shimamikan	Nagashima strain	A	1559
Miscellaneous acid citrus							
	–	*C.* sp. *tenuissima* hort. ex Tanaka	*C*. hybrid cultivar	Chosen Daidai		A	2612
	–	*C.* sp. *speciosa* hort. ex Tanaka	*C*. hybrid cultivar	Zuishoyu		A	5523
	–	*C. yuzukichi* hort. ex Y.Tanaka	*C*. hybrid cultivar	Yuzukichi		A	5513
	–	*C. kizu* hort. ex Y. Tanaka	*C*. hybrid cultivar	Taninakakizu		A	5515
	–	*C. kizu* hort. ex Y. Tanaka	*C*. hybrid cultivar	Kinosu		A	5530
	–	*C. jabara* hort. ex Y. Tanaka	*C*. hybrid cultivar	Jabara		A	5529
	–	*C. takuma-sudachi* hort. ex Tanaka	*C*. hybrid cultivar	Naoshichi		A	5506
	–	*C. acidoglobosa* hort ex Tanaka	*C*. hybrid cultivar	Matsuda Sudachi		A	5510
	–	*C. nanseiensis* hort. ex Tanaka	*C*. hybrid cultivar	Zanbo		A	5519
	–	*C*. hybrid cultivar	*C*. hybrid cultivar	Kozu		A	5516
	–	*C*. hybrid cultivar	*C*. hybrid cultivar	Nagata Kozu		A	1904
	–	*C*. hybrid cultivar	*C*. hybrid cultivar	Hedzuka Daidai		C	–
	–	*C*. hybrid cultivar	*C*. hybrid cultivar	Shibahara Sour		B	–
	–	*C*. hybrid cultivar	*C*. hybrid cultivar	Hebezu		B	–
	–	*C*. hybrid cultivar	*C*. hybrid cultivar	Genko		B	–
	–	*C*. hybrid cultivar	*C*. hybrid cultivar	Tosu		B	–

z Classification number and Latin names using Tanaka’s system [53], [54].

y Latin name using Single’s system [1].

x A: Faculty of Agriculture, Saga University, B: Saga Prefectural Fruit Tree Research Station, C: Faculty of Agriculture, Kagoshima University, and D: National Institute of Fruit Tree Science, Japan.

### Polymerase Chain Reaction Amplification and DNA Sequencing

Crude extracts from approximately 10-mm^2^ regions of leaves were prepared by incubating the leaf tissue with 100 µl of a solution containing 100 mM Tris-HCl (pH 9.5), 1 M KCl, and 10 mM EDTA, at 95°C for 20 min [31]. The primers used for polymerase chain reaction amplification of the *matK* gene were matK1F (5′-ACCGTATCGCACTATGTATC-3′) and matK1R (5′-GAACTAGTCGGATGGAGTAG-3′). Using the crude extract as template, the *matK* gene was amplified by PCR with proofreading KOD FX or KOD FX Neo DNA polymerase (Toyobo, Osaka, Japan). The amplified DNA fragments were purified using the MonoFas DNA Purification Kit I (GL Sciences, Tokyo, Japan). The primers used for sequencing of the *matK* gene were matK1F, matK2F (5′-ACGGTTCTTTCTCCACGAGT-3′), matK3F (5′-GGTCCGATTTCTCTGATTCT-3′), matK1R, matK2R (5′-AGAATCAGAGAAATCGGACC-3′), and matK3R (5′-ACTCGTGGAGAAAGAACCGT-3′). The purified DNA fragments were sequenced in both directions in an Applied Biosystems 3130 Genetic Analyzer (Applied Biosystems) with a BigDye Terminator Cycle Sequencing Ready Reaction Kit v. 3.1 (Applied Biosystems), as described previously [32]. Sequence data were submitted to DDBJ/GenBank/EBI and were assigned accession numbers ranging from AB626749 to AB626802 and from AB762316 to AB762396. The DNA templates used in this study are distinct from those used in the previous study [25].

### Phylogenetic Analyses

The maximum likelihood (ML) and neighbor-joining (NJ) methods from the MEGA (version 5.05) program [33] were used to create phylogenetic trees. The reliability of each branch was tested by bootstrap analysis with 1,000 replications. The sequences of *Zanthoxylum* sp. Clayton 15 or *Triphasia trifolia* were used as an outgroup.

## Results and Discussion

We constructed multiple sequence alignments of DNA sequences containing the *matK* gene from different accessions. The typical length of the protein-coding sequences and 3′ UTRs was 1,530 bases and 100 bases, respectively. However, some indels were present in several accessions. Because no indel was observed in the *rbcL* gene of this subfamily [25], we concluded that *matK* had a relatively fast mutation rate. The *matK* sequence used in this study was different from the partial *matK* sequence used in other published studies [16], [34], [35]. It is also different from the partial *matK* sequence used in the unpublished DNA barcoding projects. In addition, although other studies [24], [36] used the entire *matK* sequence, the number of accessions tested was small.

**Figure 1:**
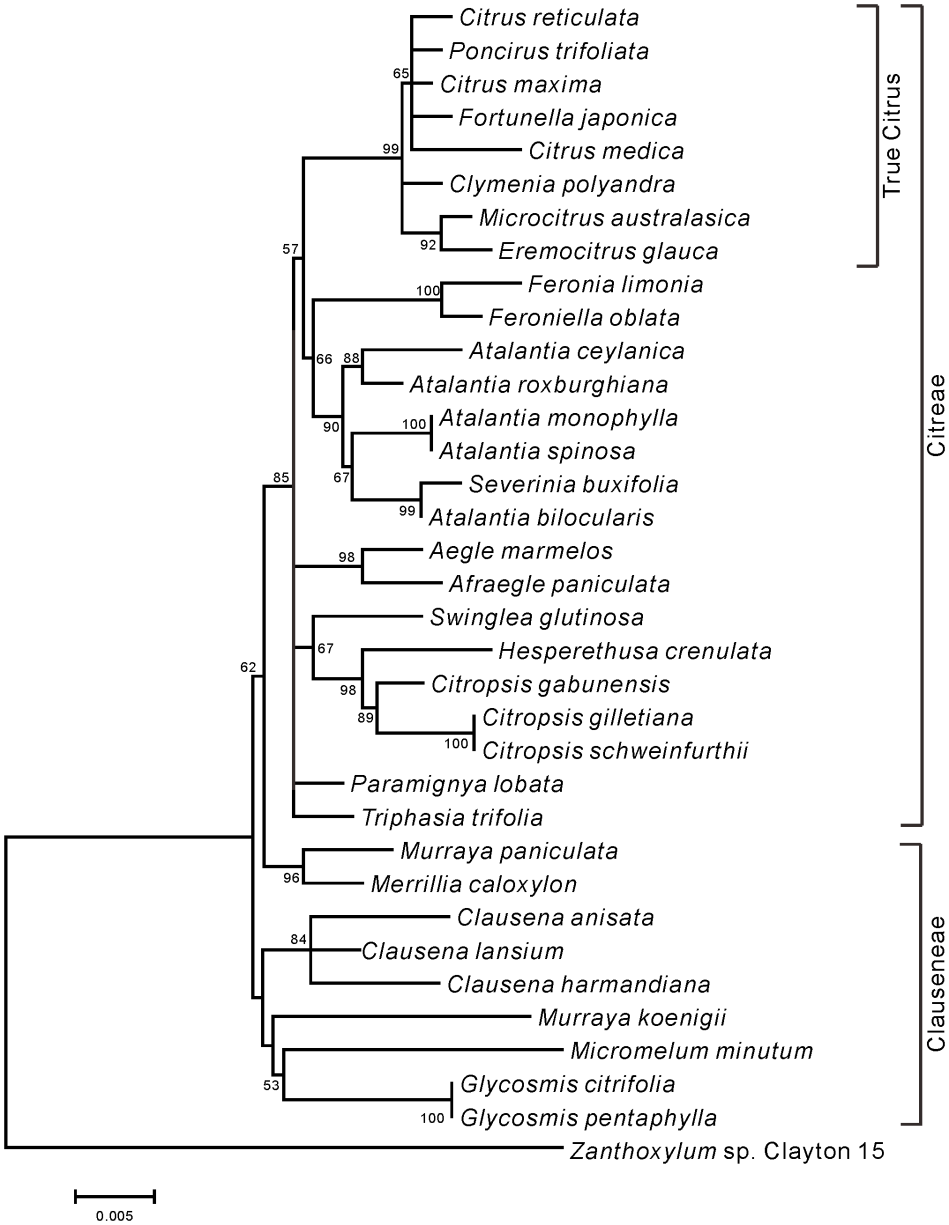
Maximum likelihood tree of the *matK* genes from accessions belonged to Aurantioideae. Numbers at the nodes indicate bootstrap values (% over 1,000 replicates).

**Figure 2:**
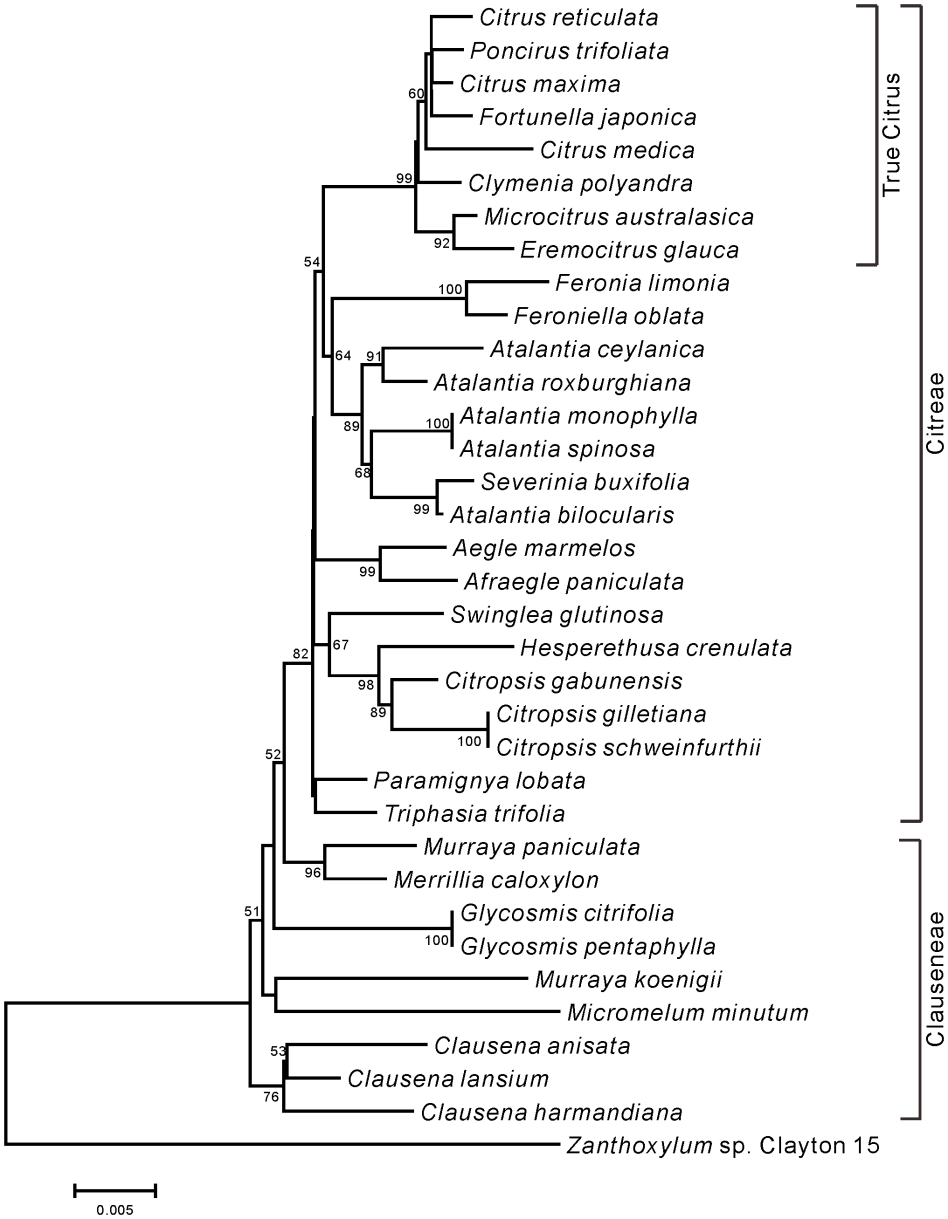
Neighbor-joining tree of the *matK* genes from accessions belonged to Aurantioideae. Numbers at the nodes indicate bootstrap values (% over 1,000 replicates).

Phylogenetic trees of Aurantioideae were created using the ML (Figure 1) and NJ (Figure 2) methods. Except for 7 species (*Glycosmis citrifolia*, *Glycosmis pentaphylla*, *Murraya koenigii*, *Micromelum minutum*, *Clausena anisata*, *Clausena harmandiana*, and *Clausena lansium*), both phylogenetic trees showed the same topology. Among these 7 species, 3 species of genus *Clausena* belonged to the same cluster, and 2 species of the genus *Glycosmis* belonged to the same cluster. A characteristic feature of both trees is that the “true citrus fruit trees” were clearly distinguished from other species. Another feature is that the “wood apple group” of Balsamocitrinae (*Feronia limonia* and *Feroniella oblata*), “primitive citrus fruit trees” (*Severinia buxifolia*) and “near citrus fruit trees” (5 species of the genus *Atalantia*) also belonged to the same cluster in both trees. The remaining species were divided into a few groups in both phylogenetic trees. One group contained “primitive citrus fruit trees” (*Hesperethusa crenulata*), “near citrus fruit trees” (3 species of *Citropsis*), and the tabog group (*Swinglea glutinosa*). Another group contained 2 species of the Bael fruit group (*Aegle marmelos* and *Afraegle paniculata*), and yet another group contained *Murraya paniculata* and *Merrillia caloxylon*. Members of the tribe Citreae belonged to the same large cluster, whereas members of the tribe Clauseneae did not.

The trees created in the present study (Figures 1 and 2) supported Swingle and Reece’s [1] classification of the subfamily Aurantioideae as monophyletic. These results are also consistent with those of Bayer et al. [16], Chase et al. [17], Groppo et al. [18], Morton et al. [20], Salvo et al. [24] and Tshering Penjor et al. [25].

The trees showed that the tribe Citreae is monophyletic, that is, they clearly discriminated Citreae from Clauseneae. This result supports Swingle and Reece’s system of tribes. Previously, Morton et al. [20] and our group [25] reported the difficulty encountered in discriminating Citreae from Clauseneae in the analyses of *rps19* and *rbcL* sequences, respectively. Our current results may be attributable to the fact that the *matK* data have high discrimination power. Although a previous study using 9 cpDNA sequences also discriminated Citreae from Clauseneae [16], we succeeded in this discrimination by using only 1 chloroplast DNA sequence. In contrast, the members of the tribe Clauseneae did not belong to the same cluster, i.e., Clauseneae is not monophyletic. Rather, the members of Clauseneae appeared to be an outgroup of Citreae. This result did not support Swingle and Reece’s system of tribes. Thus, the *matK* data only partially supported Swingle and Reece’s system of tribes. The previous study using 9 cpDNA sequences [16] also showed that the members of the tribe Clauseneae did not belong to the same cluster.

Next, we focused on Swingle and Reece’s system of subtribes. The *matK* data did not completely supported Swingle and Reece’s system of subtribes. For example, the Balsamocitrinae (*Swinglea glutinosa*, *Aegle marmelos*, *Afraegle paniculata*, *Feronia limonia*, and *Feroniella oblata*) were not placed in 1 cluster. In contrast, Swingle and Reece [1] considered that the Balsamocitrinae paralleled the Citrinae and that both had evolved from a common ancestor. Among Balsamocitrinae, the Bael fruit group of Balsamocitrinae (*Aegle marmelos* and *Afraegle paniculata*) is clustered together, which is consistent with previous reports [16], [20], [25]. Similarly, the “wood apple group” of Balsamocitrinae (*Feronia limonia* and *Feroniella oblata*) is clustered together, which is consistent with the previous reports by us [25] and Morton et al. [20].

**Figure 3:**
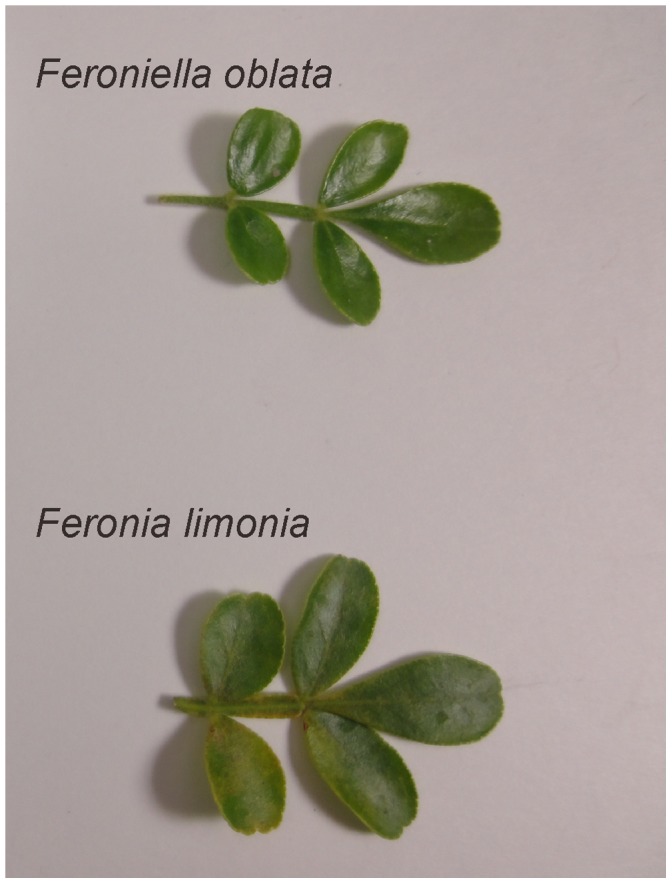
Photographs of *Feronia limonia* and *Feroniella oblata* leaves.

Interestingly, Bayer et al. [16] concluded that *Feroniella oblata* is nested in *Citrus* species. The leaves of *Feronia* and *Feroniella* are odd-pinnate with paired opposite leaflets on a rachis. In addition, they are morphologically different from other genera of the orange subfamily; the core or axis of the ovary has disappeared and an entirely new placentation has developed in this group. The 2 genera were distinguished on the basis of a few flower, seed, and fruit traits. Thus, *Feronia* and *Feroniella* are believed to be closely related to each other but they are not closely related to the remaining genera of the orange subfamily [1]. To illustrate this further we have include photographs of the leaves from these species in Figure 3. Thus, we have provided evidence that the conclusion made by Bayer et al. [16] is misleading.

In the Citrinae, our analysis did not clearly support the distinction between “primitive citrus fruit trees” and “near citrus fruit trees,” made by Swingle and Reece [1]. The tree strongly supported the polytomous clade containing *Hesperethusa crenulata* and 3 *Citropsis* species (*Citropsis gabunensis*, *Citropsis gilletiana*, and *Citropsis schweinfurthii*), which is consistent with our previous report [25]. Similarly, Morton et al. [20] reported that *Hesperethusa crenulata* and *Citropsis gilletiana* are clustered together, and Bayer et al. [20] reported that *Hesperethusa crenulata*, *Citropsis schweinfurthii*, and *Citropsis daweana* are clustered together. *Hesperethusa* and *Citropsis* have some morphological similarities such as odd-pinnate leaves with broadly winged petioles and seeds with a hard testa. In addition, both are graft compatible with *Citrus*. However, *Hesperethusa* is native to Southeast Asia, whereas *Citropsis* is present only in Africa [1].

Our matK data showed that Severinia buxifolia and 5 Atalantia species (Atalantia bilocularis, Atalantia ceylanica, Atalantia monophylla, Atalantia roxburghiana, and Atalantia spinosa) belong to a monophyletic clade. Our previous rbcL data did not lead to the same conclusions, probably because of low discrimination power [25]. Araújo et al. [15] reported that Severinia buxifolia is clustered with Atalantia monophylla, and Bayer et al. [16] reported that Severinia buxifolia is clustered with 3 Atalantia species. (Atalantia ceylanica, Atalantia citroides, and Atalantia monophylla). Our analysis showed that Severinia buxifolia is most closely related to Atalantia bilocularis. Severinia species were considered to be species of Atalantia for many years, but Swingle segregated them out in 1938 [6].

**Figure 4:**
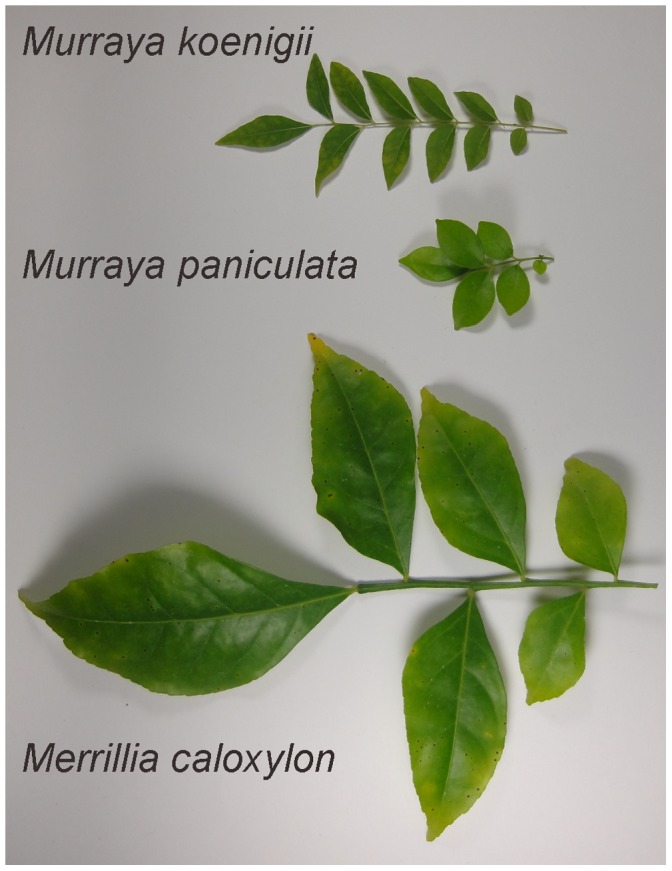
Photographs of *Merrillia caloxylon*, *Murraya paniculata*, and *Murraya koenigii* leaves.

Our *matK* data showed that *Murraya paniculata* and *Merrillia caloxylon* are clustered together, which is consistent with the previous reports by us [25] and Bayer et al. [16]. However, *Murraya koenigii* belongs to an independent cluster, which is consistent with our previous report [25]. Although the flowers of *Merrillia caloxylon*, which are very long (55–60 mm long) and trumpet shaped, are unique to the orange subfamily, this species resembles *Murraya paniculata* in its general growth habit, leaf shape, and wood texture. These 2 species grown in the wild are sometimes confused by natives of Malay Peninsula. As shown by the results from a series of studies [1], Swingle considered that *Merrillia* had probably evolved from a *Murraya*-like ancestral form. On the other hand, the leaf shapes of *Murraya paniculata* and *Murraya koenigii* are different. The leaf number of *Murraya koenigii* is larger than that of *Murraya paniculata*, although both species have odd-pinnate leaves (see Figure 4).

Our *matK* data showed that *Clausena anisata*, *Clausena harmandiana*, and *Clausena lansium* formed a monophyletic group, which is consistent with our *rbcL* data [25]. Bayer et al. [16] reported that *Clausena harmandiana* is clustered with *Clausena excavate*. Furthermore, our *matK* data showed that *Glycosmis citrifolia* and *Glycosmis pentaphylla* formed a monophyletic group, which is consistent with our *rbcL* data [25]. Bayer et al. [16] reported that *Glycosmis pentaphylla* is clustered with *Glycosmis trichanthera* and *Glycosmis mauritiana*. Thus, each of *Clausena* and *Glycosmis* forms a monophyletic group.

Both phylogenetic trees produced a well-supported (BS, 99%) clade that contained all members of the “true citrus fruit trees,” thus supporting their monophyletic origin (Figures 1 and 2). All the genera belonging to “true citrus fruit trees” were incorporated into *Citrus* in accordance with the results reported by Mabberley [37], [38], Zhang et al. [39], Bayer et al. [16], and Tshering Penjor et al. [25]. Our results support their concept.

**Figure 5:**
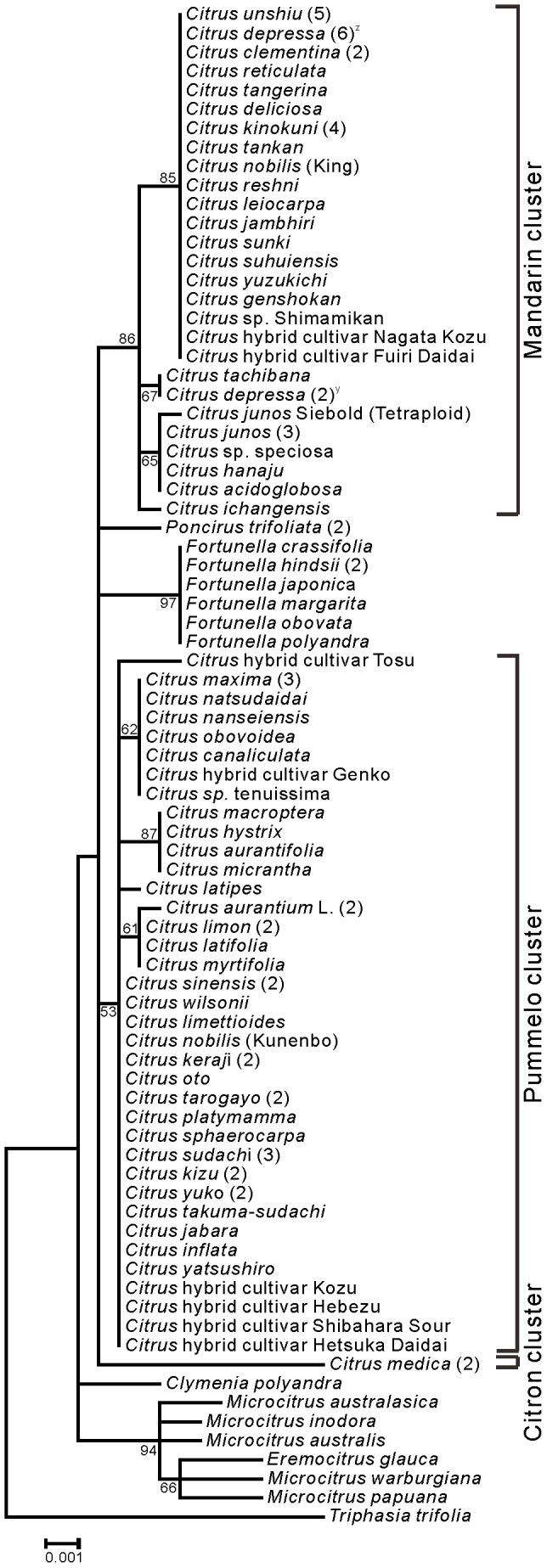
Maximum likelihood tree of the *matK* genes from accessions belonged to “true citrus fruit trees.” Numbers at the nodes indicate bootstrap values (% over 1,000 replicates). Numbers in parenthesis indicate the number of accessions. *Citrus depressa* ^z^ contains 6 accessions (Kaachi, Mikanguwa, Shiikunin, Shiikuribu, Ishikunibu, and Okitsu strains). *Citrus depressa* ^y^ contains 2 accessions (Fusubuta and Kabishi).

**Figure 6:**
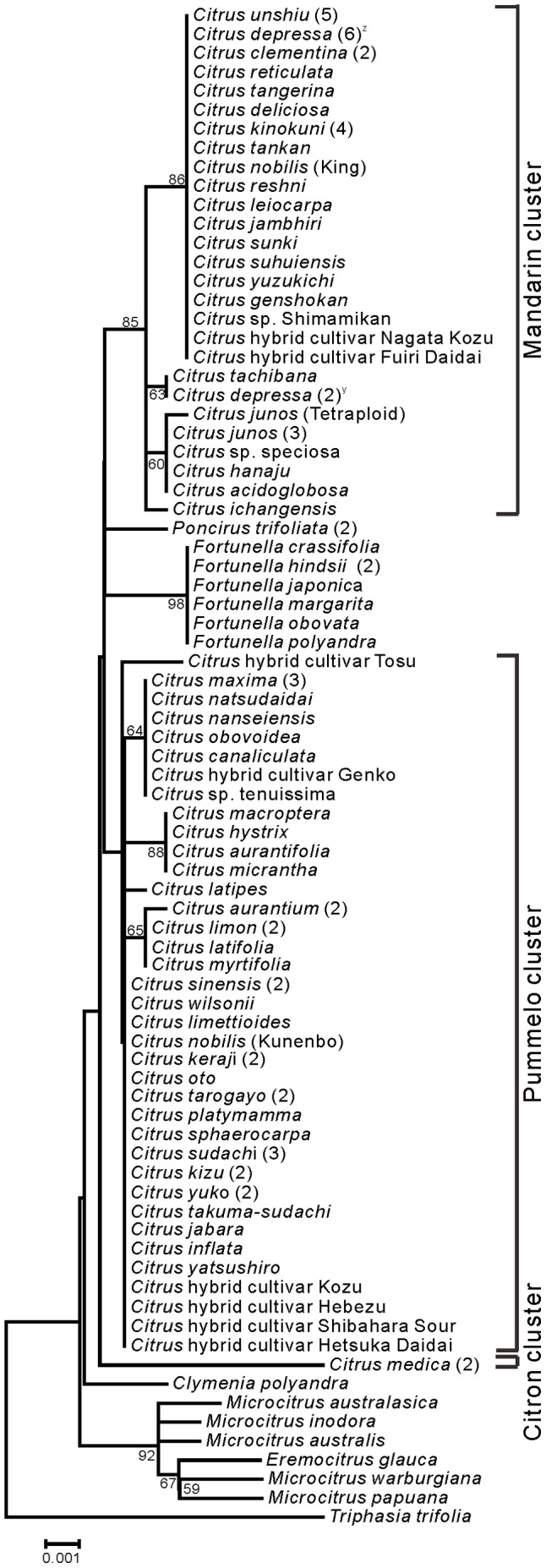
Neighbor-joining tree of the *matK* genes from accessions belonged to “true citrus fruit trees.” Numbers at the nodes indicate bootstrap values (% over 1,000 replicates). Numbers in parenthesis indicates the number of accessions. *Citrus depressa* ^z^ contains 6 accessions (Kaachi, Mikanguwa, Shiikunin, Shiikuribu, Ishikunibu, and Okitsu strains). *Citrus depressa* ^y^ contains 2 accessions (Fusubuta and Kabishi).

These “true citrus fruit trees” contain the genus *Citrus* having high economic and nutritional value. Therefore, we phylogenetically analyzed “true citrus fruit trees” by creating ML and NJ trees with *Triphasia trifolia* as an outgroup (Figures 5 and 6, respectively). The topologies of both trees were essentially identical and could be classified into 2 clusters. One large cluster included *Citrus*, *Fortunella*, and *Poncirus*. The other cluster included *Microcitrus* and *Eremocitrus*. *Clymenia* was isolated from these 2 clusters. No differences were observed among 7 *Fortunella* and 2 *Poncirus* accessions. The origins of these genera are as follows: *Citrus*, India to China; *Fortunella,* China; *Poncirus*, China; *Clymenia*, New Guinea; *Microcitrus*, Australia to New Guinea; and *Eremocitrus*, Australia. Some divergence of the *matK* sequence probably occurred between the genera originating in Southeast Asia and other places.

In the phylogenetic trees, Eremocitrus glauca was placed within the Microcitrus group (Microcitrus australasica, Microcitrus australis, Microcitrus inodora, Microcitrus papuana, and Microcitrus warburgiana). This result is consistent with Barrett and Rhodes’s results, which show a very close relationship between Microcitrus and Eremocitrus [3]. The analysis of the rbcL sequence [25] and 9 cpDNA sequences [16] also showed a close relationship between both genera. It is generally believed that the species of Microcitrus and Eremocitrus arose over millions of years of slow evolution in geographically isolated land masses (Australia and New Guinea), separate from the other genera of the true citrus group in Southeast Asia [1]. Thus, together with other studies [16], [25], our current study showed that Microcitrus species formed a monophyletic group. However, Lu et al. [23] showed that Microcitrus australasica was nested in Citrus species, although Microcitrus australis is clustered with Eremocitrus glauca. Furthermore, Li et al. [22] showed that Microcitrus australasica and Microcitrus australis are not clustered together. We suspect that the Microcitrus australasica used in the latter studies might have been of hybrid origin and that its maternal parent might be different from Microcitrus. Further study is required to confirm these conflictions.

The isolated genus *Clymenia* is native to the Bismarck Archipelago of New Guinea and is considered to be the most primitive of all the genera of “true citrus fruit trees” owing to its morphological traits [1]. Another study [16] also suggested that *Clymenia* was isolated from other species.

Our *matK* data showed that *Fortunella* species formed a monophyletic group, which is consistent with previous reports [16], [23], [25]. Swingle and Reece [1] placed *Fortunella* at the genus level only because it has 2 collateral ovules near the top of each locule, whereas *Citrus* has 4–12. However, *Fortunella* was placed within the *Citrus* group in the present study. *Fortunella* has also been classified within the *Citrus* group in previous studies on DNA analysis [10]–[14], [16], [22], [23], [25], [40], [41]. It is considered to be difficult to distinguish *Fortunella* from *Citrus* at the DNA level, based on these results.

*Poncirus* was also placed within the *Citrus* group in the present study. However, Swingle and Reece [1] placed *Poncirus* at the genus level mainly because it is deciduous, its flowering period differs from *Citrus*, it has trifoliate leaves, and its geographical distribution differs from that of *Citrus*. Several DNA analysis studies on citrus phylogeny [10], [12], [40] have revealed that *Poncirus* is distant from *Citrus*. However, some studies involving cpDNA analysis have suggested a close relationship between *Poncirus* and *Citrus* [15], [16], [20], [22], [23], [25]. These studies, along with the present study, strongly suggest that *Poncirus* is closely related to *Citrus* at the DNA level.

Next, we focused on the members of the genus *Citrus* that are economically and nutritionally important fruit trees. Our current study is the most extensive phylogenetic study of *Citrus* species among other studies (e.g., [16], [22], [23], [25]) because we studied 93 accessions of the *Citrus* species. The phylogenetic tree showed that *Citrus* can be classified into 3 clusters: the citron cluster, the pummelo cluster, and the mandarin cluster (Figures 5 and 6). This finding is consistent with the results of previous studies using not only cpDNA but also nuclear and mitochondrial DNA (mtDNA) [10]–[16], [23], [42]. We found that *Poncirus* and *Fortunella* belonged to the parent cluster containing the members of the genus *Citrus*, but did not belong to these 3 clusters.

The mandarin cluster can be divided into 1 major cluster and some minor subclusters. The major subcluster includes, amongst others, *C. reticulata*, *C. unshiu*, *C. clementina*, *C. kinokuni*, *C. deliciosa*, *C. reshni*, and *C. sunki.* One of the minor subclusters includes *C. tachibana* and *C. depressa*. The major subcluster also contains some *C. depressa* accessions. *C. ichangensis* and *C. junos* belonged to the mandarin cluster, but they were not grouped with either of the subclusters. Some Japanese acid citrus (*C.* sp. speciosa, *C. hanaju*, and *C. acidoglobosa*) were included in the *C. junos* subcluster. Similarly, Bayer et al. [16] reported that *C. reticulata*, *C. junos*, *C. tachibana*, and *C. ichangensis* were clustered together, and Lu et al. [23] reported that *C. reticulata*, *C. sunki*, and *C. tachibana* were clustered together. However, because the number of tested accessions is larger in our study, we addressed the classification of the mandarin cluster extensively, as discussed below.

Our *matK* analysis showed that most mandarin accessions belonged to the mandarin cluster. However, our extensive analysis showed that there are some exceptions. The mandarin accessions, *Citrus keraji*, *Citrus oto*, *Citrus tarogayo*, *Citrus platymamma*, and *Citrus yatsushiro* belonged to the pummelo cluster, and not to the mandarin cluster. Interestingly, *Citrus nobilis* (King) belonged to the mandarin cluster, and *Citrus nobilis* (Kunenbo) belonged to the pummelo cluster. Similarly, the previous study [22] showed that most mandarin landraces formed a monophyletic clade and some exceptions were similar to pummelo, although their tested accessions were different from ours.

The major subcluster of the mandarin cluster includes edible mandarins such as *C. reticulata*, *C. unshiu*, and *C. clementina*. In the present study, it was difficult to further classify members of this subcluster. Similarly, the previous researchers who conducted analyses based on cpDNA [12], [22], [43], [44] and mtDNA [14], [41] did not subdivide these members. Thus, our analysis confirmed the similarities of these mandarins with respect to organellar DNA. The major subcluster of the mandarin also includes *C. reshni*, *C. sunki*, and *C. depressa*, which are small-fruited mandarins mainly used as rootstocks. In contrast, the previous study using 3 cpDNA sequences [23] separated *Citrus sunki* from *Citrus reticulata*. One of the minor subclusters of the mandarin consists of *C. tachibana* and *C. depressa*, which are native to Japan. Hence, *C. tachibana* and some *C. depressa* accessions are different from other members of the mandarin cluster. This finding is consistent with the findings of previous studies that used cpDNA analyses [12], [15], [19], [23], [40], [43]. Furthermore, the analyses of mtDNA [14], isozymes [6], and chromosomes [30] also separated *C. tachibana* from the other members of the mandarin cluster. Thus, our analysis confirms that *C. tachibana* differs from other members of the mandarin cluster. Members of *C. depressa* are placed in both the major and the minor subclusters. Consistent with our analysis, the analyses by Urasaki et al. [44] and Yamamoto et al. [45] showed the genetic diversity in cpDNA among the members of *C. depressa*. These results suggest that different *C. depressa* accessions have different origins.

The pummelo cluster contained, amongst others, *C. maxima*, papeda, *C. sinensis*, *C. limon*, and *C. aurantifolia*. As described above, this cluster also contained some mandarin accessions such as *C. nobilis* (Kunenbo) and *C. keraji*. This cluster can be divided into 7 subclusters, which are represented by the species *C. sinensis*, *C. maxima*, *C. latipes*, *C. aurantifolia* (plus papeda), *C. aurantium*, and *C. limon* and the *Citrus* hybrid cultivar ‘Tosu’. A number of mandarins and Japanese acid citrus belonged to the *C. sinensis* subcluster. Similarly, Bayer et al. [16] reported that *C. sinensis*, *C. maxima*, *C. aurantifolia*, papeda, *C. aurantium*, and *C. limon* were clustered together. However, because the numbers of tested accessions are larger in our study, we addressed the classification of the pummelo cluster extensively, as discussed below.

Some of the mandarins are clustered exclusively with pummelo accessions. This result strongly suggests that their maternal origins are members of the pummelo cluster. Previous studies showed that *C. nobilis* (Kunenbo) has organellar DNA derived from a member of the pummelo cluster [14], [46]. Its maternal predecessor is probably a sweet orange with cytoplasmic DNA originally derived from a member of the pummelo cluster. *C. nobilis* (Kunenbo) is thought to be the maternal ancestor of *C. keraji* (Keraji and Kabuchii) and *C. oto* [46], [47]. Consistent with that hypothesis, the results of the current study show that the *matK* sequences from *C. keraji* (Keraji and Kabuchii), *C. oto*, and *C. nobilis* (Kunenbo) are identical. In agreement with the results of a previous morphological characterization [48], the results of our analysis suggest that *C. nobilis* (Kunenbo) is also the maternal origin of *C. tarogayo*.

We also studied the nucleotide variation in the *matK* genes from accessions that were grouped in the pummelo cluster, although the diversity is low. By using SSR analysis, Deng et al. [43] showed that cpDNAs of pummelos and their relatives have many types of nucleotide polymorphisms. Except for pummelo (*C. maxima*) and papeda, all members of the pummelo cluster originate from hybrids. Therefore, the nucleotide variation among the maternal ancestors can contribute to the variation in the pummelo cluster. Our results show that the *matK* sequences of *C. aurantium*, *C. sinensis*, *C. limon*, and *C. limettioides* are derived from members of the pummelo cluster, and this finding is consistent with the results of previous studies using cpDNA analysis [12], [15], [16], [40], [47], [49]. *C. wilsonii* and *C. latifolia* also have pummelo-type cpDNA sequences, and to our knowledge, this is the first study to highlight this point. The *matK* sequences from *C. aurantium* and *C. limon* in the pummelo cluster differ by single-base pair mismatches. Previous analyses of cytoplasmic DNA suggested that *C. aurantium* is the maternal ancestor of *C. limon* [14], [50]. Thus, further work is required to confirm this result.

Swingle and Reece [1] divided the genus *Citrus* into the subgenera *Citrus* and *Papeda*. However, in the present study, papeda belonged to the pummelo cluster, and it is difficult to discern the subgenera *Citrus* and *Papeda*. Thus, our analysis does not support the classification proposed by Swingle and Reece [1]. Similar results were reported based on the cpDNA analyses [12], [16]. The *matK* sequences from *C. hystrix*, *C. micrantha*, and *C. macroptera* are identical, which is consistent with the results of previous analyses of cpDNA by Nicolosi et al. [12]. They suggested that *C. micrantha* was the maternal ancestor of *C. aurantifolia*. Our present results show that the *matK* sequence from *C. aurantifolia* is identical to that from *C. hystrix*, *C. micrantha*, and *C. macroptera*, which confirms the results of the study by Nicolosi et al. [12]. However, the *rbcL* genes and chloroplast SSRs of *C. micrantha* and *C. aurantifolia* differ [25], [43]. Therefore, their maternal relationship is unclear.

Most of miscellaneous acid citrus species grown in Japan belonged to the mandarin major cluster, the *C. junos* cluster, or the *C. sinensis* cluster. Although this result almost agrees with that of Asadi Abkenar et al. [51] who analyzed cp and mtDNA using PCR-RFLP, our analysis showed that *matK* sequences of some accessions (*C.* sp. *tenuissima* and *C. nanseiensis*) were identical with that of *C. maxima*. As above mentioned, *matK* sequence of *C. sinensis* was similar to that of *C. maxima* but not identical with it. Our result was more informative than the previous study [51] and it is considered to be useful information for estimation of genetic background of Japanese acid citrus. These data obtained from cytoplasmic DNA analysis were not coincident with the RAPD data [40]. Furthermore, our analysis clearly separated *Citrus* hybrid cultivar ‘Tosu’ from the other citrus. Thus, these accessions appear to have arisen from complicated cross and combined results of nuclear and cytoplasmic genomes; further studies are required to elucidate their exact their origin.

Swingle and Reece [1] have classified *C. ichangensis* into the subgenera *Papeda*. However, our analysis shows that *C. ichangensis* belongs to the mandarin cluster. There are conflicting reports regarding the cytoplasmic relationships between *C. ichangensis* and other *Citrus* species. Consistent with our current result, based on the analyses of cpDNAs, Asadi Abkenar et al. [40], Bayer et al. [16], Deng et al. [43], and Nicolosi et al. [12] showed that *C. ichangensis* is closely related to the mandarins. However, according to the phylogeny of *rbcL* [25], *C. ichangensis* is most closely related to the *Poncirus* species. On the other hand, cpSSR analysis [49] failed to show a relationship to other species. A comparison of mtDNA sequences [41] suggested that *C. ichangensis* was not related to the mandarins and was identical to *C. hystrix* and *C. aurantifolia*. A complete understanding of the cytoplasmic relationships of *C. ichangensis* with the *Citrus* species and its relatives including the *Poncirus* species requires further analyses.

Our analysis clearly separated citron from pummelo and mandarin. Considerable variation between citron and the other *Citrus* accessions has been reported previously based on Fraction I protein [52], mtDNA [14] and cpDNA [12], [15], [16], [22], [23], [42].

## Conclusions

Based on the chloroplast *matK* sequences, the present study provides novel information that resolves the genetic relationships among members of the Aurantioideae, especially of the genus *Citrus,* and confirms previous observations. Because the *matK* gene has a relatively fast rate of nucleotide substitutions, our study provides more information on interspecific relationships within the genus *Citrus* than the analysis of the *rbcL* gene [25]. Our extensive classification of 135 accessions from 22 genera of the Aurantioideae could be useful for the breeding of these trees.
